# Increasing Severity of Spinal Cord Injury Results in Microglia/Macrophages With Annular-Shaped Morphology and No Change in Expression of CD40 and Tumor Growth Factor-β During the Chronic Post-injury Stage

**DOI:** 10.3389/fnmol.2021.802558

**Published:** 2022-02-24

**Authors:** Elvira Ruslanovna Akhmetzyanova, Anna Viktorovna Timofeeva, Davran Khudaishukurovich Sabirov, Alexander Alexandrovich Kostennikov, Alexander Alexandrovich Rogozhin, Victoria James, S. S. Arkhipova, Albert Anatolevich Rizvanov, Yana Olegovna Mukhamedshina

**Affiliations:** ^1^Clinical Research Center for Precision and Regenerative Medicine, Institute of Fundamental Medicine and Biology, Kazan Federal University, Kazan, Russia; ^2^Department of Neurology, Kazan State Medical Academy–Branch Campus of the Federal State Budgetary Educational Institution of Father Professional Education, Russian Medical Academy of Continuous Professional Education, Kazan, Russia; ^3^Division of Biomedical Science, Faculty of Medicine and Health Sciences, School of Veterinary Medicine and Science, University of Nottingham Biodiscovery Institute, University Park, Nottingham, United Kingdom; ^4^Department of Histology, Cytology and Embryology, Kazan State Medical University, Kazan, Russia

**Keywords:** spinal cord injury, microglia, rat, polarization, morphology

## Abstract

Determination of the quantitative composition of phenotypically and morphologically different populations of resident microglia and infiltrating macrophages in spinal cord injury (SCI) of various degrees of severity could lead to much needed novel therapeutic interventions in neurotrauma. In this regard, we investigated the CD40 and TGF-β expressing populations of microglia/macrophages and their morphological states in a rat model of SCI of varying severity. We are the first to describe the annular-shaped microglia/macrophages, the morphology of which was formed due to the spatial orientation of the processes that form round or oval micro-territories, which include disintegrating myelin fibers. This type of cell morphology was found only in the injured spinal cord and mainly in the white matter. At the same time, an assessment of the number of annular-shaped microglia/macrophages and the diameter of micro-territories formed by their processes showed an elevation in these indicators as the severity of SCI increased. While we did not find significant quantitative changes in the populations of Iba1^+^/CD40^+^ and Iba1^+^/TGF-β^+^ microglia/macrophages with increased severity of SCI in the chronic period (60 dpi), we did determine changes in the expression of cytokines and mRNAs of genes-encoding microglial marker proteins, finding the greatest changes on days 7 and 14 after SCI between experimental groups with varying severity.

## Introduction

Numerous studies over the past decades have undeniably demonstrated the importance of the microglia in the pathogenesis of central nervous system (CNS) injuries (Kawabori and Yenari, 2015; Devanney et al., 2020). A key feature of microglia is their ability to change their phenotype and morphology under the influence of the microenvironment, a process known as polarization (Hu et al., 2015). This process determines a wide range of responses of microglia, including changes in their phagocytic activity and the secretion of various cytokines (Zhou et al., 2014; Akhmetzyanova et al., 2018; Bellver-Landete et al., 2019).

Microglia are described based on their polarization, neurotoxic (M1), and neuroprotective (M2) phenotypes of these cells that are distinguished, which have a specific effect on the microenvironment. In the case of microglia activation and acquiring the M1 phenotype, it is supposed that neuroinflammatory damage is triggered, mediated by the production of pro-inflammatory cytokines such as interleukin-1β (IL-1β), tumor necrosis factor-α (TNF-α), superoxide, nitric oxide, and reactive oxygen species (ROS) (Block et al., 2007; Qiu et al., 2020). It has previously been described that an alternative variant of microglia activation leads to polarization toward the M2 phenotype, which antagonizes the pro-inflammatory response and has a positive effect on the outcome of neurotrauma through the secretion of IL-4, IL-13, IL-10, and tumor growth factorβ (TGF-β) (Butovsky et al., 2005; Zhou et al., 2012; Chhor et al., 2013). Nevertheless, the progress of research in recent years has given us grounds for revising the above terminology (Ransohoff, 2016). Transcriptomic analysis has shown a substantial diversity of expression profiles of microglia and infiltrating macrophages, which were not concordant with the M1 and M2 phenotypes in accordance with a continuum model of polarization (Xue et al., 2014; Wes et al., 2016). Due to these data and more recent studies using single-cell RNA sequencing (scRNAseq), new terminology is starting to be introduced, such as disease-associated microglia (DAM) (Keren-Shaul et al., 2017; Deczkowska et al., 2018; Hammond et al., 2019), which, in our opinion, also does not fully reveal the various roles and functions of microglia.

The behavior of microglia and their phenotypic traits have been studied in detail in various brain lesions (Loane and Kumar, 2016; Xu et al., 2017; Lyu et al., 2021). However, our knowledge of microglia in spinal cord injury (SCI) is not yet sufficient. Bellver-Landete et al. (2019) reported different populations of microglia/macrophages are present in the area of spinal cord damage within the 1st week post injury in mice, with the number of M1 cells exceeding the number of M2. This absence of M2 microglia/macrophages was also found in the chronic period post-injury (28 days), as well as a significant decline in M1 microglia when compared to 7 days post-injury (Kigerl et al., 2009; Francos-Quijorna et al., 2016). A more recent study by Hakim et al. (2021) revealed stable cellular reprogramming of resident microglia into DAM, beginning 3 days post-SCI, which strongly enhanced recovery.

To date, there is no accepted classification and terminology of the morphological forms of microglia, which are distinguished by a significant diversity among glial cells. This is exemplified in the varying published reports, which predominantly describe the diverse morphologies of microglia as ramified, ramified/hyper-ramified, de-ramified, orbipolar/rod morphologies (Morrison et al., 2017; Holloway et al., 2019), while other published studies refer to fried egg, ameboid and hypertrophic morphologies of microglia (Lin et al., 2017; Gao et al., 2018; Zhang et al., 2020; Banerjee et al., 2021). It should be noted that the morphology of microglia does not always indicate their phenotype. Thus, it was shown that the population of bipolar/rod-shaped microglia *in vitro* have a high proliferative potential and express markers characteristic of both M1 (TNF-α, IL-1b, CD32, and CD86) and M2 (IL-10 and TGF- β) phenotypes (Tam and Ma, 2014). However, other comparisons of amoeboid and bipolar/rod-shaped microglia have found that amoeboid cells produce significantly higher levels of pro-inflammatory cytokines more akin to an M1 phenotype. Furthermore, Kigerl et al. (2009) described M1 microglia/macrophages as cells with numerous short, highly branched processes, and M2 phenotype as cells with single long processes in a mouse model of SCI.

Despite the available data, it has not yet been established whether changes in the quantitative composition of phenotypically and morphologically different populations of microglia affect the severity of SCI. The potential correlation between the nature and intensity of polarization of microglia in a certain direction and the outcome of SCI may serve as the basis for the development of a new direction of therapeutic intervention, the search for which in neurotrauma is particularly relevant, given the current lack of effective treatment strategies.

## Materials and Methods

### Spinal Cord Contused Injury

All animal protocols were approved by the Kazan Federal University Animal Care and Use Committee (Permit Number: 2, dated on May 5, 2015). Adult female Wistar rats weighing 250–300 g each were obtained from Pushchino Laboratory, Russia. Animals were housed 3–4 per cage and kept in 12-h light/dark cycle with food and water available *ad libitum*.

Surgeries were conducted with isoflurane anesthesia and intramuscular injection of Zoletil (20 mg/kg, Virbac Sante Animale, France) as the analgesia. After skin incision, the Th8 vertebra was removed by laminectomy. The impact rod of an impactor was centered above Th8 and dropped to induce SCI of varying degrees of severity: light (1.5 m/s, *n* = 20), moderate (2.5 m/s, *n* = 20) and severe (4 m/s, *n* = 20) (Impact One Stereotaxic Impactor, Leica). Additionally, 36 rats were operated on in each group to collect spinal cord samples at days 3, 7, and 14 after SCI to carry out cytokine analysis and Real-Time PCR. Intact animals (*n* = 15) were used as our control group. All postoperative procedures were performed according to the previously described protocol (Mukhamedshina et al., 2017).

### Behavioral Study

Motor function was evaluated using the open-field Basso, Beattie, Bresnahan (BBB) locomotor rating scale (Basso et al., 1995). The BBB scale rates locomotor assessment and ranges from 0 to 21. A baseline was obtained 3 days before SCI. Open-field locomotor testing was carried out one time a week. To evaluate differences in functional recovery, a behavioral assessment in the control and experimental groups was performed before SCI. Locomotion was scored simultaneously by 2 trained investigators who were blinded to the treatment groups.

### Electrophysiological Studies

Electrophysiological tests were performed on intact rats and control/experimental rats 2 and 11 weeks post injury (wpi) as previously described (Mukhamedshina et al., 2019). Compound Muscle Action Potential (CMAP) and H-response were recorded from the gastrocnemius muscle with monopolar needle electrodes. An active electrode was placed in the middle of the muscle belly, in the region of the Achilles tendon. Monopolar needle electrodes inserted subcutaneously within an area where the sciatic nerve exits from the pelvis were used for stimulation. Electrical stimulation of the sciatic nerve was carried out with square-wave single stimuli lasting for 0.2 ms.

Transcranial electrical stimulation was used for evaluation of central motor pathways. Motor evoked potentials (MEPs) were registered from gastrocnemius muscle by the same technic as CMAP. Needle electrodes were inserted under the scalp in contact with the bone of the skull to activate the motor cortex. The cathode was positioned in the midline approximately 0.5 cm caudally from the interorbital line, with the anode on the midline near the occipital bone. Stimuli duration was 0.04 ms, with variations in intensity from 20 to 400 V.

Two-channel recording of somatosensory evoked potentials (SEPs) was used to evaluate the central sensory pathways. Registration was done with monopolar needle electrodes inserted subcutaneously. Registration from the lumbar level: an active electrode over upper lumbar vertebras, a referent electrode—over middle thoracic vertebras. Registration from scalp: an active electrode—in the midline approximately 0.5 cm caudally from the interorbital line, a referent electrode—in the midline near the occipital bone. Electrical stimulation of tail performed by superficial round electrodes with stimulus duration of 0.2 ms. Stimulus intensity was chosen by the threshold of tail movements (the lowest stimulus to produce tail movements was used).

### Histological Assessment

For histology and immunohistochemistry, day 60 postinjury (dpi) rats were euthanized and intracardially perfused with 4% paraformaldehyde (PFA, Sigma). After overnight incubation in 4% PFA, spinal cords were removed and placed in 15% sucrose for 24–48 h. Before slicing, spinal cords were stored in 30% sucrose. Non-fixed tissue was used for RT-PCR. Then, samples were frozen in a tissue freezing medium (Tissue-Tek O.C.T. Compound, Sakura Finetek). About 20- μm transverse tissue slices were sectioned on a Microm HM 560 cryostat. After Azur-eosin staining, tissues were visualized using a 20× objective (APERIOCS2, Leica). The cross-sectional area of spared tissue and abnormal cavities was measured on transverse sections of the spinal cord within the midpoint of the lesion center (the epicenter) and in spinal segments 1–5-mm rostral and caudal to the site of injury. A total area of abnormal cavities in the spinal cord cross-section was calculated by adding cysts with an area of not less than 1.500 μm^2^. Aperio imagescope software was used for measuring the tissue area.

### Immunoelectron Microscopy

For immunoelectron microscopy, spinal cord samples isolated at a distance of 5 mm caudally from the Th8/epicenter of the SCI were fixed in 10% PFA mixed with 0.2% phosphate-buffered glutaraldehyde solution (Alfa Aesar, Thermo Fisher Scientific) at 4°C for 12 h, with post-fixation in 0.5% OsO4 (Sigma) in PBS for 1 h at room temperature (RT). After fixation, the samples were dehydrated in an ethanol gradient and encapsulated in LR White polymer resin (Electron Microscopy Sciences). A Leica UC7 ultramicrotome (Leica) was used to obtain ultra-thin sections of 0.1-μm thickness. Ultrathin sections were mounted on nickel grids coated with a formvar film. Slices were then blocked with TBS-NDS-BSA-TX-100 [Tris buffer solution (Tris.01 M, NaCl.15-M pH = 8.2)], 10% normal donkey serum, 0.2% bovine serum albumin, and Triton X-100 0.1% for 1 h at RT. After washing in TBS, sections were incubated overnight at 4°C with antibodies against Iba1 (Supplementary Table 1). For immune gold labeling, sections were washed and incubated with colloidal gold-conjugated secondary antibodies (Supplementary Table 1) for 2 h at RT.

For immune peroxidase labeling, sections were incubated in 0.5% H_2_O_2_ for 30 min, followed by 60 min in 1% H_2_O_2_, and then again for 30 min in 0.5% H_2_O_2_. Sections were then rinsed with PBS and incubated free-floating in Iba1 antibody with 3% normal horse serum, 0.05% Triton-X in PBS, for 24 h rotating at RT. The tissue was then rinsed in 3 wells for 30 s in PBS and incubated for 1 h in biotinylated universal antibody (1:200, Vector Labs), rotating at RT. Then, the sections were rinsed in 3 wells for 30 s in PBS and incubated for 1 h in ABC (Vector Labs) solution, rotating at RT. Following incubation, sections were rinsed with PBS for 3 min and were developed by incubating in 0.025% diamino-benzidine (DAB) and 0.002% hydrogen peroxide in PBS. The DAB reaction was halted by the addition of PBS, followed by 3- x-30-s washes in PBS.

Then, obtained sections were stained with uranyl acetate and lead citrate for contrast and evaluated using a transmission electron microscope (Hitachi HT7700). The ZEN blue Lite program and software provided with the Hitachi 7,700 transmission electron microscope were used to measure the size of gold nanoparticles as well as the filament thickness.

### Immunohistochemical Studies

After blocking in PBS containing 5% normal goat serum for 1 h at RT, the tissue slices were incubated overnight at 4°C with primary antibodies (Abs) (Supplementary Table 1). Prior to visualization, the sections were incubated with corresponding fluorophore-conjugated secondary Abs (Supplementary Table 1) for 2 h at RT and counterstained with 4′,6-Diamidino-2-phenylindole (DAPI) (10 μg/ml in PBS, Sigma) to visualize the nuclei. The sections were examined using an LSM 780 Confocal Microscope (Carl Zeiss). A quantitative count of cells expressing TGF-β, Iba1, and CD40 was carried out. All sections were imaged in the z-plane using identical confocal settings (laser intensity, gain, and offset). Measurements were obtained from transverse histological sections collected at a distance of 5 mm caudally from the epicenter of SCI. The following areas were selected for immunohistochemical analysis of microglia: the ventral horn (VH), the main corticospinal tract (CST), and ventral funiculi (VF).

### Study of Microglia Morphology

Microglia shape and size in the experimental groups were assessed by fractal analysis using FracLac for ImageJ, which quantifies a span ratio and density as previously described (Morrison et al., 2017). Four annular-shaped microglia were chosen for fractal analysis within each photomicrograph (5 photomicrographs per animal) within the VF and VH zone. Due to the fact that we did not find annular-shaped microglia in the intact spinal cord, ramified microglia were selected as a comparison population, the span ratio and density of which were analyzed using the above method. We also quantified annular-shaped microglia and estimated the diameter of micro-territories formed by their processes in the VF and VH zone of the experimental groups.

### Obtaining Homogenates of Spinal Cord Tissue

To evaluate the spinal cord cytokine profile in experimental animals, a portion of the spinal cord 5 mm in length at the site of injury/Th8 was prepared and homogenized with an electric homogenizer in 300 μl of a complete extraction buffer. The blade was rinsed two times with 300 μl of a complete extraction buffer, and constant agitation was maintained for 2 h at 4°C. After centrifugation for 20 min at 15,000 rpm at 4°C, a soluble protein extract was collected and frozen at −80°C until the cytokine multiplex assay was performed.

### Cytokine Assay

Multiplex analysis based on the xMAP Luminex technology was performed following the manufacturer’s instructions for the bead-based immunoassay Bio-Plex Pro Rat Cytokine Group l Panel 23-plex Assay #12005641 (Bio-Rad). Experiments were performed in triplicate. The kit enables a simultaneous multiplex analysis of 23 cytokines, chemokines, and interleukins in a 50- μl sample.

### RNA Isolation, cDNA Synthesis, and Real-Time PCR

Total RNA was isolated from fresh spinal cords (a 5-mm long segment encompassing the injury site) using Yellow Solve Kit (Silex, Russia) according to the manufacturer’s instructions. RNA quality and quantity were measured using a NanoDrop (Thermo Scientific). About 100 ng of total RNA was reverse transcribed using a 100 U of RevertAid reverse transcriptase (Thermo Scientific), 100 pmol of random hexamer primers, and 5 U of the RNAse inhibitor according to the recommended manufacturer’s protocol (25°C–10 min, 42°C–60 min, termination of transcription, 70°C–10 min). A quantitative analysis of mRNA of Iba1, IL-6, TGF-β, CD209, and CD40 genes was performed using a CFX 96 Real-Time PCR System (Bio-Rad). Amplification conditions were as follows: 95°C–3 min, 39 cycles: 95°C–10 s, 55°C–30 s, including plate read. Each reaction was performed in duplicate in a total volume of 10 μl and contained 100 ng of diluted cDNA, 2.5 × Reaction mixture B (Syntol), 200 nM of each primer and 100-nM SybrGreen (Eurogen) (Supplementary Table 2). The mRNA expression was normalized using GAPDH rRNA. Plasmid DNA with corresponding inserts was used to create standard curves for quantification. To assess copy number of the plasmid DNA insert, we used DNA copy number calculator1. Cq range for 18S was 13–14 cycles, while the test genes ranged from 25 to 31.

### Statistical Analysis

Results generated were expressed as a mean ± SD. ANOVA with Tukey’s test was used for multiple comparisons between all investigated groups. Mann-Whitney test was used to analyze the results of the PCR analysis. All analyses were performed in a blinded manner with respect to the treatment group. The mean differences among the experimental groups were considered significant if *p* < 0.05 was reached. Data were analyzed using Origin 7.0 SR0 Software (OriginLab).

## Results

### Structural and Functional Changes Under Conditions of Rat Spinal Cord Injury’s Varying Degrees of Severity

**FIGURE 1:**
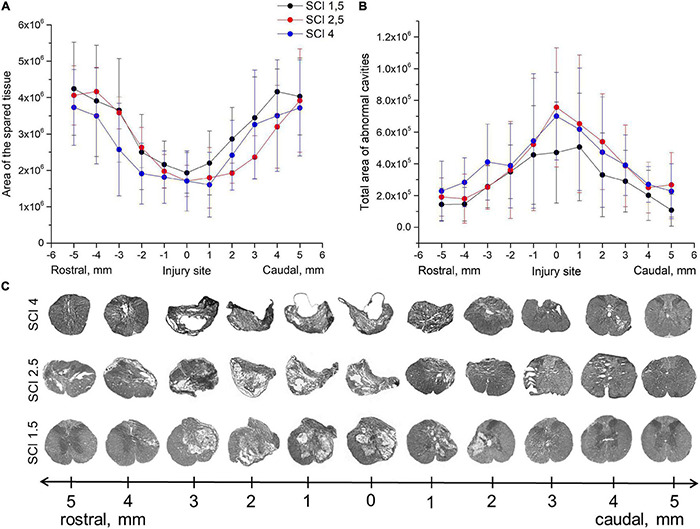
Tissue analysis in experimental groups. An area of the spared tissue **(A)** and a total area of abnormal cavities **(B)** 5 mm rostrally and caudally from the injury epicenter on Day 60 after spinal cord injury of mild (SCI 1.5, *n* = 8), moderate (SCI 2.5, *n* = 8), and severe (SCI 4, *n* = 8) severities. *No significant differences in the above indicators of morphometric analysis were found between the experimental groups, one-way ANOVA followed by a Tukey’s post hoc test.* **(C)** Cross sections of the injured spinal cord on Day 60 after spinal cord injury in experimental groups with azur-eosin staining.

Morphometric analysis showed that the area of the spared tissue at 60 dpi was greater in the rostral direction (1–5 mm) from the epicenter of the injury ingroups where the SCI was light (SCI 1.5) or medium (SCI 2.5) in severity compared to the group with the most severe injury (SCI 4) and in the caudal direction in the light SCI 1.5 group compared to the medium SCI 2.5 and severe SCI 4 groups (Figures 1A–C). The area of abnormal cavities was minimal at all studied distances from the epicenter of injury in the SCI 1.5 group compared to the more severely injured SCI 2.5 and SCI 4 groups (Figures 1B,C). At the same time, a higher value of abnormal cavities area was noted at a distance of 3–5 mm rostrally in the SCI 4 group compared to SCI 2.5 and SCI 1.5 groups. Nevertheless, no significant differences in the above indicators of morphometric analysis were found between the experimental groups.

**FIGURE 2:**
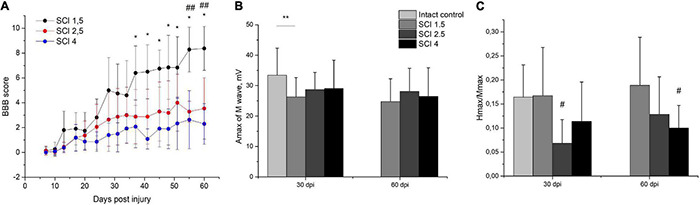
Post-SCI behavioral and electrophysiological studies in experimental groups. Assessment of locomotor activity using the Basso, Beattie, Bresnahan (BBB) rating scale from 7 to 60 days after spinal cord injury of mild (SCI 1.5), moderate (SCI 2.5), and severe (SCI 4) severities **(A)**, *n* = 20 in each group. The motor function scores were highest in the SCI 1.5 group compared to SCI 2.5 and SCI 4 groups, starting from 2 wpi. *p < *0.05 as compared with the SCI 4 group; ^##^p* < *0.05 as compared with the SCI 2.5 group, one-way ANOVA followed by a Tukey’s post hoc test.* Investigation of sciatic nerve conduction on 30 and 60 dpi was shown by Amax of the M-wave **(B)** and H/M wave amplitude ratio **(C)** in experimental groups. At 30 dpi, the amplitude of the M-response in the SCI 1.5 group and the Hmax/Mmax in the SCI 2.5 group was lower compared to the intact controls. At 60 dpi, differences in the Hmax/Mmax were only observed between the SCI 4 group and the intact control. **p < *0.05;*^#^*p* < *0.05 as compared with intact control, one-way ANOVA, followed by a Tukey’s post hoc test.*

The locomotor activity of hind limbs was carried out using the BBB behavioral test from 7 to 60 dpi. The motor function scores were highest in the SCI 1.5 group compared to SCI 2.5 and SCI 4 groups, starting from 2 wpi (Figure 2A). At the same time, significant differences between SCI 1.5 and SCI 2.5 groups were seen during the last week of the experiment, and between SCI 1.5 and SCI 4 groups at 37–60 dpi. It should be noted that the hind limb locomotor activity scores in the SCI 2.5 group were higher compared to the SCI 4 group within 60 dpi, but without significant differences.

Investigation of sciatic nerve conduction on 30 dpi revealed a decrease (*p* < 0.05) in the amplitude of the M-response of the gastrocnemius muscle in the SCI 1.5 group as compared with the intact controls (Figure 2B). At the same time, no differences in this indicator between the experimental groups were noted either at 30 or 60 dpi. On 30 dpi, the H-reflex was recorded as significantly less frequent in the SCI 2.5 group compared to the intact control and SCI 1.5 group. In the same period, the Hmax/Mmax was lower (*p* < 0.05) in the SCI 2.5 group compared to the intact controls (Figure 2C). At 60 dpi, there were no differences in the frequency of the H-response between the experimental groups and intact controls. However, the Hmax/Mmax was lower in the SCI 4 group compared to the intact controls.

Investigation of the central motor pathways by the method of transcranial electrical stimulation in all experimental groups at 30 and 60 dpi demonstrated there was a pronounced decrease in the frequency of registration and the amplitude of gastrocnemius muscle MEPs during stimulation of the cortex (Table 1). About 60-dpi MEPs were significantly more frequent in the SCI 1.5 group (in 60% of cases) compared with the SCI 2.5 group (in 30% of cases) and the SCI 4 group (in 25% of cases). It should be noted that there were no differences in the amplitude characteristics of MEPs in the experimental groups.

**TABLE 1:** Results of transcranial electrical stimulation and motor evoked potential (MEP)/scalp somatosensory evoked potential (SEP) registration in rats.

Groups	Lack of MEPs in both legs (in% of Rats)	Registration of MEPs from one leg (in No. of rats)	Lack of SEPs (in% of Rats)	Registration of SEPs (in% of Rats)
Intact control	–	–	–	100
SCI 1.5 30/60 dpi	50/40	–	5/5	95/95
SCI 2.5 30/60 dpi	65/70	–	5/0	95/100
SCI 4 30/60 dpi	80/75	–	45/10	55/90
				

When evaluating the central somatosensory pathways in all animals within the intact control group, SSEP peaks were recorded from electrodes on the lumbar spine (lumbar peaks) and electrodes placed under the scalp skin (cortical peaks). Lumbar peaks after SCI continued to be recorded in all animals in the experimental groups without differences in amplitude, including when compared with the intact control group, which reflected the preservation of the peripheral part of the somatosensory pathway. At the same time, in the experimental groups, there was a significant decrease in the frequency of cortical SSEP peaks compared with the intact control group (Table 1). At the same time, on 30 dpi in the SCI 4 group, cortical peaks were recorded more often compared to SCI 1.5 and SCI 2.5 groups, but, on 60 dpi, these differences between the experimental groups disappeared.

Summarizing the obtained results, we can conclude that the SCI 1.5 group had a significant improvement in the central motor pathways at 60 dpi, which made it possible to maintain normal excitability of the reflex arc at the lumbar level, in contrast to the SCI 2.5 and SCI 4 groups.

### Cytokine Profile in Spinal Cord Tissue

**FIGURE 3:**
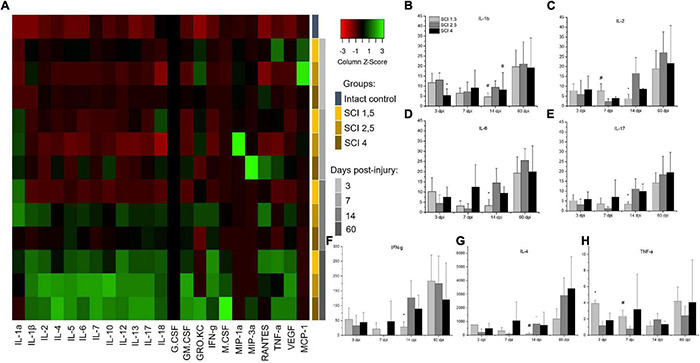
Analysis of the cytokine profile of contused and intact spinal cords. Heat map analysis of the cytokine profile (color keys) in the spinal cords of experimental groups and intact controls **(A)**. Concentration (Y axis, pg/ml) of IL-1b, IL-2, IL-6, IL-17, IFN-g, IL-4, and TNF-a at days 3, 7, 14, and 60 after spinal cord injury of mild (SCI 1.5), moderate (SCI 2.5), and severe (SCI 4) severities was analyzed **(B–H)**, *n* = 7 in each group and each of the above dpi. The cytokine expression levels in the intact spinal cord were considered as 1. *p < *0.05 as compared with all investigated groups;*^#^*p* < *0.05 as compared with the SCI 1.5 group, one-way ANOVA, followed by a Tukey’s post hoc test.*

To determine the levels of cytokine expression in the spinal cord following injury of varying degrees of severity, multiplex immunoassays were performed. Significant differences were found in the content of some analytes in the acute (3–7 dpi) and subacute (14 dpi) periods after injury (Figure 3A). The level of the pro-inflammatory cytokine IL-1β was significantly increased in all SCI injury groups compared to the intact controls (*p* < 0.05) by 3 dpi. At the same time, in the group of animals with severe trauma (SCI 4), IL-1β was lower (*p* < 0.05) compared with the less severe SCI 1.5 and SCI 2.5 groups (Figure 3B). On 14 dpi, the expression of IL-1β in the spinal cord of animals with mild trauma (SCI 1.5) showed a gradual decrease, showing a significant difference with the medium severity SCI 2.5 group (8.8 ± 3.7 vs. 33.3 ± 17.9, respectively). Similarly, the level of pro-inflammatory cytokine IL-2 also increased in all SCI groups (except on days 3 and 7 post-injury in the SCI 2.5 group) when compared to the intact controls (*p* < 0.05). However, significant differences in IL-2 expression were observed between the experimental groups at 7 and 14 dpi (Figure 3C). At 7 dpi, the highest IL-2 level was observed in the mild trauma group (SCI 1.5), with a significant difference from the SCI 2.5 group (177.5 ± 85.4 vs. 52.6 ± 30.1, respectively). However, at 2 wpi, the situation changed dramatically with IL-2 levels significantly decreased in the SCI 1.5 group and increased by more than 2 times in the more severely injured SCI 2.5 and SCI 4 groups [85.5 ± 52.8 (SCI 1.5) vs. 382.1 ± 183.7 (SCI 2.5) and 198 ± 5.9 (SCI 4), *p* < 0.05].

A similar trend on 14 dpi was found for other pro-inflammatory cytokines IL-6, IL-17, and IFN-g, the levels of which were significantly lower during this period in the group with mild trauma (SCI 1.5) when compared with the more severely injured SCI 2.5 and SCI 4 groups (Figures 3D–F). At the same time (14 dpi), the concentration of IL-4 and IL-12 in the SCI 1.5 group was also minimal, but significant differences were found when compared to the SCI 2.5 group [IL-4 –0.8 ± 1 (SCI 1.5) vs. 8.3 ± 5.3 (SCI 2.5), *p* < 0.05; IL-12–25.7 ± 14.2 (SCI 1.5) vs. 82.7 ± 34.2 (SCI 2.5), *p* < 0.05] (Figure 3G). However, on 14 dpi, pro-inflammatory cytokines IL-1a and GRO/KC were increased in the mild trauma group (SCI 1.5) compared to the SCI 4 group (*p* < 0.05). It is also noteworthy that, in the same period, the levels of anti-inflammatory cytokine IL-10 and angiogenic/neurotrophic factor VEGF were, significantly, the lowest in the SCI 1.5 group.

TNF-a expression in spinal cord was also measured. At 3dpi, the highest expression of TNF-a was found in the mild trauma SCI 1.5 group [218.6 ± 20.7 vs. 64.1 ± 34.1 (SCI 2.5) and 101.9 ± 49.1 (SCI 4), *p* < 0.05]. Subsequently, there was a gradual decrease in the TNF-a level in this group (SCI 1.5), where at 14-dpi TNF-a expression was at its lowest when compared to the dynamic of TNF-a expression in the other experimental groups [62.2 ± 36.5 vs. 106.9 ± 41.6 (SCI 2.5) and 74.5 ± 21.3 (SCI 4)] (Figure 3H). A similar dynamic was established for IL-5, the level of which was highest on days 3 and 7 post-injury in the SCI 1.5 group, but, at 14 dpi, became significantly lower than in the other experimental groups [21.6 ± 6.6 vs. 44.7 ± 13.8 (SCI 2.5) and 43.7 ± 15.1 (SCI 4)].

It should be noted that some of the cytokines underwent significant changes in the group with moderate injury (SCI 2.5). Thus, on 3 and 7 dpi, the pro-inflammatory cytokine IL-18 showed a lower (*p* < 0.05) level of expression in the area of damage in animals of the SCI 2.5 group compared to the other experimental groups. Similar changes on 3 dpi were noted for RANTES, where the level of this analyte in the SCI 2.5 group was significantly lower compared to the SCI 1.5 and SCI 4 groups by 5- and 7-fold, respectively. At the same time, the expression of chemokine MCP-1 was the highest in the SCI 2.5 group, showing a significant difference to the SCI 4 group at 3 dpi. The level of MCP-1 at 7 dpi in the SCI 2.5 group remained the highest (∼ three times higher, *p* < 0.05) compared to other experimental groups.

### mRNA Expression of Microglia-Specific Genes

**FIGURE 4:**
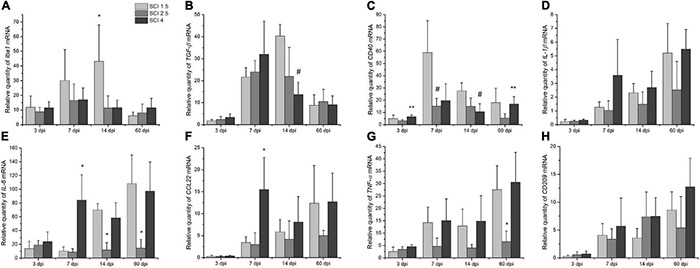
Analysis of mRNA expression in the area of SCI in rats. Relative quantity of *Iba1, TGF*-β, *CD40, IL-1*β, *IL-6, CCL22, TNF*-α, *and CD209* mRNA expression **(A–H)** at 3, 7, 14, and 60 days after spinal cord injury of mild (SCI 1.5, a light gray column), moderate (SCI 2.5, a gray column), and severe (SCI 4, a black column) severities (*n* = 5 in each group and each of the above dpi), calculated in relation to intact spinal cord controls, which were considered as 1. The mRNA expression was normalized using GAPDH rRNA. *p < 0.05 as compared with all investigated groups; **p < *0.05 as compared with the SCI 2.5 group; ^#^p* < *0.05 as compared with the SCI 1.5 group, one-way ANOVA, followed by a Tukey’s post hoc test.*

The expression of *Iba1, IL-6, IL-1b, CCL22, TNF-a, TGF-*β, and *CD209* mRNAs were established in the intact and injured rat spinal cord groups on days 3, 7, 14, and 60 post injury. The highest expression of *Iba1* mRNA was noted in the group with mild trauma (SCI 1.5) from 3 to 14 dpi with a maximum value in the subacute period (14 dpi), where a significant difference was found compared to the other experimental groups [43.1 ± 24.7 vs. 11.3 ± 8.2 (SCI 2.5) and 11.6 ± 4.9 (SCI 4)] (Figure 4A). On 60 dpi, there was a significant decrease in the expression of *Iba1* mRNA in the SCI 1.5 group, from which point the expression of *Iba1* was no longer significantly different from groups SCI 2.5 and SCI 4 but remained increased in relation to the intact controls. Similar results were obtained for *TGF-*β mRNA levels, which increased to a greater extent at 7 and 14 dpi with the maximum value in the SCI 1.5 group by 14 dpi, where it was ∼3-fold higher (*p* < 0.05) when compared to the SCI 4 group (Figure 4B). Similarly to *Iba1* expression, the level of *TGF-*β was reduced at 60 dpi in the mild trauma SCI 1.5 group, with no significant difference to *TGF-*β expression in the other experimental groups, although *TGF-*β expression remained elevated relative to the intact controls.

The level of *CD40* mRNA expression was at its highest in the mild trauma SCI 1.5 group at 7 dpi, where its value was ∼4-fold higher (*p* < 0.05) than the levels found in the SCI 2.5 group (Figure 4C). At 14 and 60 dpi, a gradual decrease in the expression of *CD40* mRNA was observed in the SCI 1.5 group, the level of which was not significantly different from the other experimental groups. In the acute (3 dpi) and chronic (60 dpi) periods, we also found significant differences in the expression of *CD40* between the groups with moderate and severe injury groups, with higher expression in the SCI 4 group.

Similar to *CD40, IL-1β* and *IL-6* mRNA expression was significantly increased in the severe trauma (SCI 4) group at 7 dpi; these levels decreased slightly at 14 dpi, but increased again by 60 dpi, with maximum expression values observed in both SCI 1.5 and SCI 4 groups at 60 dpi (Figures 4D,E). It should be noted that the level of *IL-6* mRNA expression was ∼100-fold higher (*p* < 0.05) in the SCI 1.5 and SCI 4 groups on 60 dpi compared to the intact control, which is consistent with the results obtained by multiplex analysis of IL-6 protein levels.

The level of *CCL22* mRNA was also increased at 7 dpi, with the highest expression found in the group with severe trauma (SCI 4) [15.4 ± 7.2 vs. 3.4 ± 1.3 (SCI 1.5) and 3 ± 2.6 (SCI 2.5), *p* < 0.05] and a subsequent decrease by 14 dpi (Figure 4F). As with *IL-6*, the level of *CCL22* mRNA at 60 dpi also increased again in the SCI 1.5 and SCI 4 groups. The greatest changes in the expression of *TNF-a* mRNA also concerned the groups with mild (SCI 1.5) and severe trauma (SCI 4), in which this mRNA gradually increased from 3 to 60 dpi and was significantly higher compared to the SCI 2.5 group (Figure 4G). Finally, expression of *CD209* mRNA expression gradually increased from 3 to 60 dpi, but differences in expression did not reach significance between the experimental groups at the investigated periods after SCI (Figure 4H).

### Study of Changes in Microglia Morphology

**FIGURE 5:**
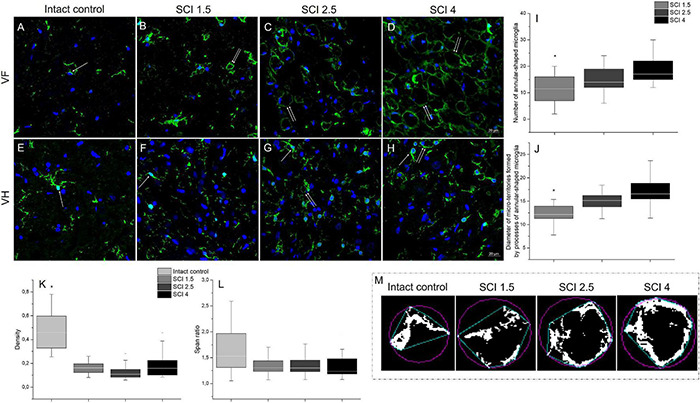
Analysis of microglia morphology in SCI and intact controls. Visualization of microglia morphology using anti-Iba1 antibody (green) within the ventral funiculi (VF) and ventral horn (VH) at 60 days after spinal cord injury of mild (SCI 1.5; **B,F)**, moderate (SCI 2.5; **C,G)**, and severe (SCI 4; **D,H**) severities and intact controls **(A,E)** at a distance of 5 mm caudally from the epicenter of injury. All sections were imaged in the z-plane using identical confocal settings (laser intensity, gain, and offset). Annular-shaped microglia indicated by the arrow. Nuclei are DAPI-stained (blue). Scale bar: 20 μm. Number of annular-shaped microglia **(I)** and diameter of micro-territories formed by processes of annular-shaped microglia **(J)** within the VF at 60 days after SCI 1.5 (a light gray column), SCI 2.5 (a gray column), or SCI 4 (a black column). *p < *0.05 as compared with all investigated groups, one-way ANOVA, followed by a Tukey’s post hoc test.* Summary data of microglia density **(K)** and span ratio **(L)** within the VF at 60 days in abovementioned experimental groups and intact controls. Schematic illustration of the convex hull (blue) and bounding circle (purple) in binary photomicrographs in the investigated groups necessary for calculating the microglia span ratio and density **(M)**.

Microglia morphology was analyzed at 60 dpi at a distance of 5 mm caudally from the epicenter of the SCI in the different experimental groups. In addition to the characteristic and previously described morphologies of microglia (ramified and ameboid), we also found annular-shaped microglia in the white matter in the analyzed VF and CST zones. The morphology of which was formed due to the spatial orientation of the processes that form round or oval micro-territories (Figures 5A–D). It should be noted that such microglia morphology was rarely observed in the gray matter; annular-shaped microglia were in single figures in this zone (Figures 5E–H).

In the VF zone, where we observed the largest abundance of annular-shaped microglia, we analyzed the number and diameter of micro-territories formed by their processes. In the group with severe damage (SCI 4), the number of annular-shaped microglia was the highest [18.85 ± 5.5 vs. 11.14 ± 5.1 (SCI 1.5, *p* < 0.05) and 14.86 ± 4.6 (SCI 2.5)], and the diameter of micro-territories formed by their processes reached 16.86 ± 3 μm, which was 32.1% more (*p* < 0.05) than in the SCI 1.5 group (12.76 ± 2.7) (Figures 5I,J).

We analyzed microglia morphology related to cell shape: span ratio and density. The microglia span ratio and density in the area of damage in the experimental groups were reduced by 30.7% and ∼3-fold (*p* < 0.05), respectively, when compared with the intact controls (Figures 5K–M). However, there were no significant differences in these parameters between the different severities of SCI.

**FIGURE 6:**
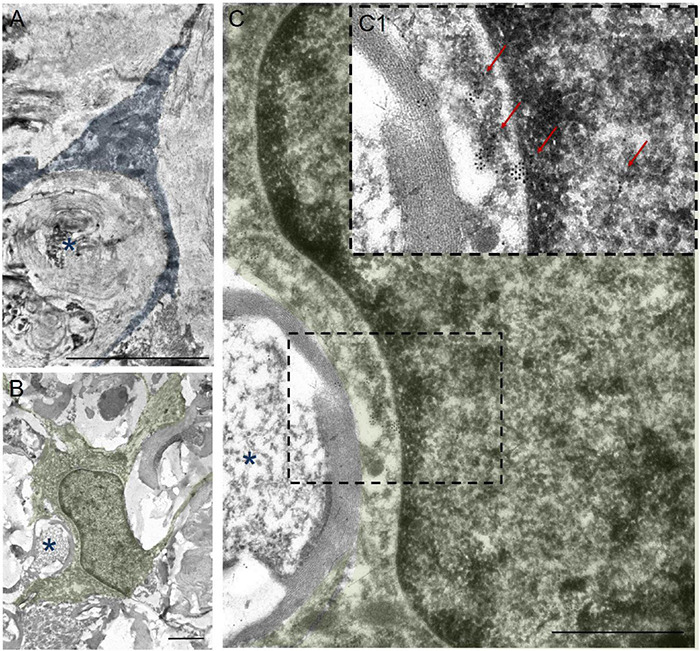
Ultrastructure of annular-shaped microglia in SCI. Visualization of microglia ultrastructure using anti-Iba1 antibody within the ventral funiculi (VF) at 60 days after a mild contusion injury (the SCI 1.5 group). Positive immuno-peroxidase staining with anti-Iba1 antibodies in the cytoplasm of the microglia processes (marked in blue) surrounded disintegrating myelin fibers (asterisks) **(A)**. Positive immunocytochemical reaction with anti-Iba1 antibodies conjugated with gold nanoparticles (arrows) in the nucleus, perikarion, and processes of the microglia (marked in green) **(B,C,C1)**. The detected annular-shaped microglia had an increased electron density of the cytoplasm; the organelles were poorly visualized, but mitochondria with indistinctly distinguishable crystae were detected. The **(C)** area marked with dashed boxes corresponds to enlarged images of **(C1)**. Obtained sections evaluated using a transmission electron microscope (Hitachi HT7700). Scale bar: **(A)** 5, **(B)** 2, and **(C)** 1 μm.

In addition to the above, we performed immunoelectron microscopic analysis in order to understand which structures are surrounded by the processes of annular-shaped microglia. Our results indicate that disintegrating myelin fibers are present in round or oval micro-territories formed by the processes of annular-shaped microglia (Figure 6). Disintegrated myelin forms concentric multilayer electron-dense structures. Immunoperoxidase staining using anti-Iba1 antibody demonstrates a suspended electron density in the cytoplasm of the processes located between adjacent areas of disintegrated myelin and surrounding those areas with thinner processes (Figures 6A,B). Immunocytochemical staining with gold nanoparticles showed the expression of Iba1 in both the cytoplasm and the nucleus of microglia (Figures 6C,C1). Accumulations of gold nanoparticles were also visualized between the layers of disintegrated myelin (Figure 6C1). The detected annular-shaped microglia had an increased electron density of the cytoplasm; the organelles were poorly visualized, but mitochondria with indistinctly distinguishable crystae were detected. Our data confirm the change in microglia cell shape/elongation and size and the existence of the annular-shaped form of these cells, which surround disintegrating myelin fibers after SCI.

**FIGURE 7:**
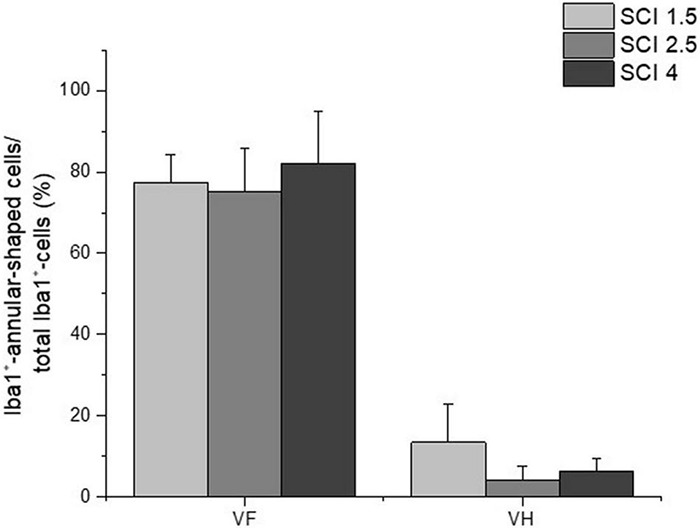
The percentage ratio of Iba1^+^-annular-shaped microglia in SCI. The percentage ratio of Iba1^+^-annular-shaped cells to the total number of Iba1^+^-cells (Y-axis) in the ventral funiculi (VF) and ventral horn (VH) at 60 days after spinal cord injury of mild (SCI 1.5, a light gray column), moderate (SCI 2.5, a gray column), and severe (SCI 4, a black column) severities at a distance of 5 mm caudally from the epicenter of injury. *No significant differences in the above indicator of morphometric analysis were found between the experimental groups, one-way ANOVA, followed by a Tukey’s post hoc test.*

The study also analyzed the percentage ratio of Iba1^+^-annular-shaped cells to the total number of Iba1^+^-cells in the experimental groups, which was higher in ∼10 folds in the VF compared to the VH zone (Figure 7). However, there were no significant differences within each analyzed area for percentage of Iba1^+^-annular-shaped cells among the experimental groups with different degrees of SCI severity.

### Quantitative Changes in CD40 and Tumor Growth Factor-β Expressing Microglia Populations

**FIGURE 8:**
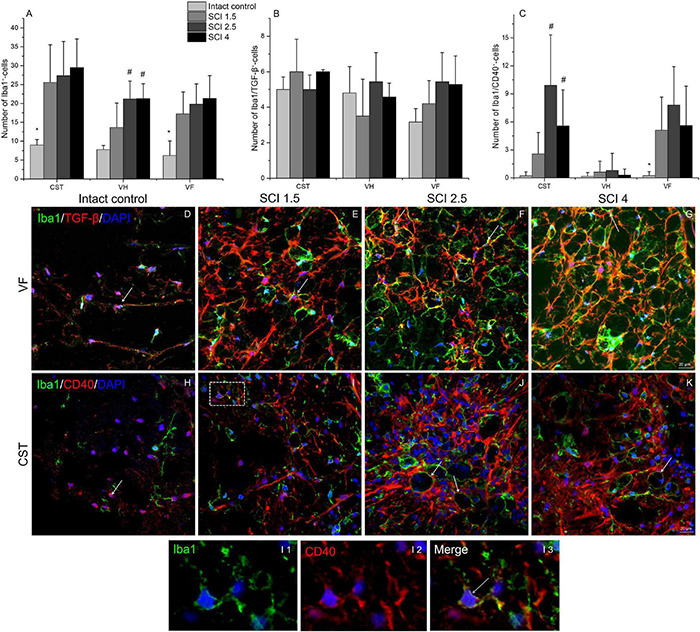
Quantitative changes in CD40 and TGF-β expressing microglia populations. The number of Iba1^+^ **(A)**, Iba1^+^/TGF-β^+^ **(B)**, and Iba1^+^/CD40^+^ cells **(C)** (Y-axis) in the examined regions (X-axis) of the intact spinal cord and experimental groups with spinal cord injury of mild (SCI 1.5), moderate (SCI 2.5), and severe (SCI 4) severities (*n* = 8 in each group). Visualization of different microglia populations using Iba1 (green), TGF-β (red) or CD40 (red) 5 mm caudally from the injury epicenter (60 dpi) within the ventral funiculi (VF) and the main corticospinal tract (CST) in the investigated groups **(D–K)**. Iba1^+^/TGF-β^+^ and Iba1^+^/CD40^+^ cells indicated by the arrow. All sections were imaged in the z-plane using identical confocal settings (laser intensity, gain, and offset). The I area marked with dashed boxes corresponds to enlarged images I1, I2, and I3. Nuclei are DAPI stained (blue). Scale bar: 20 μm. *p < *0.05 as compared with all investigated groups; ^#^p* < *0.05 as compared with intact control, one-way ANOVA, followed by a Tukey’s post hoc test.*

The total number of microglia was estimated using the pan marker Iba1. The results showed that, on 60 dpi, the number of Iba1^+^cells remained elevated in the experimental groups compared to the intact controls (Figure 8A). However, no significant differences in the number of Iba1^+^ microglia were found in the VF, VH, or CST zones when comparing the experimental groups with different degrees of SCI severity.

The number of different microglia populations after SCI of varying degrees of severity was assessed by immunohistochemical analysis to determine Iba1^+^/TGF-β^+^ (M1 neurotoxic) and Iba1^+^/CD40^+^ (M2a neuroprotective) cells (Walker and Lue, 2015). Iba1^+^/TGF-β^+^cells, in contrast to Iba1^+^/CD40^+^ cells, were more evenly distributed in the injured spinal cord, and no significant quantitative differences were found in the VF, VH, or CST compared to controls (Figures 8C, H–K). At 60 dpi, both the number of Iba1^+^/TGF-β^+^ and TGF-β^+^ cells at a distance of 5 mm caudal from the epicenter of the injury did not undergo significant changes, regardless of the severity of the SCI.

The number of Iba1^+^/CD40^+^cells increased in the SCI groups compared to the intact controls in both the CST [0.22 ± 0.4 (Intactcontrol) vs. 9.91 ± 5.6 (SCI 2.5) and 5.58 ± 3.8 (SCI 4), *p* < 0.05] and VF regions [0.23 ± 0.4 (Intactcontrol) vs. 5.11 ± 3.5 (SCI 1.5) and 7.81 ± 4.1 (SCI 2.5) and 5.6 ± 4.2 (SCI 4), *p* < 0.05] with the maximum number in the moderate severity SCI 2.5 group (Figures 8, H–K). However, we again did not find significant changes in the number of Iba1^+^/CD40^+^ cells in the investigated areas between the groups with SCI of varying degrees of severity.

The study also analyzed the percentage ratio of Iba1^+^/TGF-β^+^ and Iba1^+^/CD40^+^ cells to the total number of Iba1^+^-cells in the experimental groups (Table 2). The highest percentage of Iba1^+^/TGFβ^+^ cells was found in the intact control group compared to the SCI experimental groups. In the VF, the percentage of Iba1^+^/TGFβ^+^ cells was significantly lower in the VF region in the mild trauma SCI 1.5 group and the VH region in the severe SCI 4 group. In all three experimental groups, the number of Iba1^+^/TGFβ^+^ cells was lower in the CST region. At the same time, the percentage of Iba1^+^/CD40^+^cells increased in the SCI groups in the CST and VF regions, where it was no less than 5.5-fold higher (*p* < 0.05) in the experimental groups compared to the intact controls.

**TABLE 2:** The percentage ratio of Iba1^+^/TGF-β^+^ and Iba1^+^/CD40^+^ cells compared to all populations of Iba1^+^ cells in intact and contused spinal cord in rats at 60 dpi.

Groups	Iba1^+^/TGF-β^+^cells (in%)			Iba1^+^/CD40^+^cells (in%)		
	VF	VH	CST	VF	VH	CST
Intact control	49.5 ± 13.8	54.7 ± 16.6	55.9 ± 7.4	4.8 ± 9.2	2.7 ± 5.8	4.9 ± 11.6
SCI 1.5	21.7 ± 7.4	34.2 ± 24.1	19.9 ± 7.3	37.1 ± 22.6	3 ± 5.7	12.3 ± 7.5
SCI 2.5	30.6 ± 11.7	26.8 ± 9.4	26 ± 3	39.4 ± 24.4	4.2 ± 9.8	11.54 ± 7.9
SCI 4	27.2 ± 9.7	22.1 ± 6.1	25.4 ± 0.6	26.2 ± 16.6	1.6 ± 3.5	18.5 ± 13.6

*The underlined data indicate significance has been reached (p < 0.05) when compared to the intact control group.*

## Discussion

Although there are a large number of studies on the pathogenesis of SCI, the molecular and cellular mechanisms underlying the reprogramming of microglia toward a neurotoxic and/or neuroprotective phenotype are still unclear. To date, it has not been established which factors and intercellular interactions play the greatest role in the polarization of microglia in one direction or another. This is confirmed by the fact that, in the presence of a significant arsenal of investigated inhibitors of microglia activity, not one of the options has yet found its application through clinical trials. A possible contribution to the development of the dichotomy of responses from the microglia is the degree of nervous tissue injury. However, until now, such studies have not been carried out, although they can potentially be of great importance not only for fundamental science but also for the practical application and development of an optimal therapeutic strategy.

The abovementioned confirms the high significance and relevance of our research on the quantitative and qualitative assessment of various populations of microglia in the rat model of SCI of varying degrees of severity. Before proceeding with the direct study of microglia, we had validated the contusion rat SCI model of mild, moderate, and severe injuries. The obtained data on morphometric analysis are consistent with Noble and Wrathall (1985), who showed that, with an increase in SCI severity, the area of spared tissue decreases. We also found using BBB scoring that motor recovery decreased with increasing severity of SCI. Barros Filho and Molina (2008) showed that the BBB score has high reproducibility for mild SCI and lower reproducibility for moderate and severe injuries. This finding explains the absence of a significant difference between the moderate SCI 2.5 and severe injury SCI 4 groups in our study. We obtained similar behavioral data by functional assessment using electrophysiological studies. Thus, the group with mild severity of injury (SCI 1.5) had an advantage over the groups with moderate and severe SCI, since it had significantly higher conduction indices in the central motor pathways by 60 dpi, maintaining the normal excitability of the reflex arc at the lumbar level. These results are consistent with the study by Cao et al. (2005), where the authors showed that, with an increase of SCI severity, the spared tissue decreases, and motor functions and nerve fiber conductivity deteriorate. This study as well as ours noted that a significant difference in the abovementioned parameters between groups with moderate and severe degrees of damage is not always present. However, in general, this comprehensive approach to assessing the structure and function of the spinal cord in SCI of varying degrees of severity have made it possible to confirm the creation of various conditions for the restoration of nerve tissue.

As well as quantitative and qualitative assessment of various populations of microglia, we believe that the assessment of the cytokines that make up the microenvironment in the SCI region is also of great importance and can clarify the reasons for the reprogramming of microglia toward a neurotoxic and/or neuroprotective phenotype. In our study, we found the levels of both pro-inflammatory and anti-inflammatory cytokines at 3 dpi significantly increased compared to the intact controls, while often reaching maximum values at 60 dpi. Earlier, it was shown that, despite the chronic period, microglia produce a huge amount of pro-inflammatory cytokines, which corresponds to the course of the second wave of microglia activation (Noble and Wrathall, 1989; Milich et al., 2019). It should be noted that no significant differences in the expression of cytokines between the experimental groups were observed at 60 dpi. It can be assumed that, in the chronic period (60 dpi), post-traumatic processes proceed in a similar way, regardless of the severity of the injury. Nevertheless, we cannot assert that only microglia make a contribution in the composition of the cytokine profile in the damaged area.

Using RT-PCR, we found an increase in the level of *Iba1* mRNA in SCI, which is a logical consequence of the course of the primary posttraumatic processes and recruitment of microglia (Poulen et al., 2021). At the same time, we observed the greatest increase of *Iba1* and *TGF*-I mRNA levels on 14 days after mild SCI. Indirectly, this may indicate an increase in the number of neuroprotective microglia and more significant suppression of immune processes in the case of mild SCI in the subacute period (Morino et al., 2008). The *CCL22* mRNA level, the expression of which, as well as *TGF*-β, is characteristic of neuroprotective microglia (Jurga et al., 2020), which also showed the most significant differences between the experimental groups in the early period after SCI (7 dpi), where the maximum value was noted in the group of animals with severe SCI damage. At the same time, the *CD40* mRNA level, the expression product of which is an early marker of neurotoxic microglia activation (Ponomarev et al., 2006), had a consistently higher level of expression in the mild trauma SCI 1.5 group also in the early period after injury compared to other experimental groups. Thus, we found the greatest increase and significant changes of *Iba1, TGF-β*, and *CD40* mRNA expression between groups with varying degrees of SCI severity on 7 and 14 dpi. At the same time, the expression of *IL-1β, IL-6, TNF-α*, and *CD-209* mRNA gradually increased after SCI and reached their maximum expression at 60 dpi. These data indicate that inflammatory processes in different periods after SCI are most likely mediated by different mechanisms, which, during the chronic period (60 dpi), then proceed in a more similar manner, regardless of injury severity.

In the experimental groups, mainly in the white matter of the VF and CST regions, we found annular-shaped microglia, the morphology of which was formed due to the spatial orientation of the processes that form round or oval micro-territories, which surrounded disintegrating myelin fibers. Surprisingly, we did not find any previous description of such microglia morphology arising in SCI. Furthermore, we have established for the first time that the number of annular-shaped microglia and the diameter of micro-territories formed by their processes is directly related to the degree of damage—as the severity of SCI increases. Overall, our data confirm the change in microglia cell shape/elongation and size and the existence of the annular-shaped form of these cells.

To assess the different populations of microglia/macrophages in the SCI area, we used immunohistochemical analysis of Iba1^+^/TGF-β^+^ and Iba1^+^/CD40^+^ cells. When assessing the total population of Iba1^+^ microglia in the injured spinal cord compared to intact controls, we found a significant increase by 60 dpi. While no significant differences in Iba1^+^-microglia were observed between the experimental groups, these results, corresponding to the increase of Iba1^+^-microglia in injured spinal cord of rats in the delayed chronic period, are consistent with the works of others (Wu et al., 2005). However, for the first time, we also carried out a quantitative assessment of the neuroprotective (Iba1^+^/TGF-β^+^ cells) and neurotoxic (Iba1^+^/CD40^+^ cells) populations of microglia during this period. On 60 dpi, the number of Iba1^+^/TGF-β^+^ cells in the investigated areas of the experimental groups did not undergo significant changes and were present at similar levels to those seen in the intact spinal cord controls. Nevertheless, the percentage ratio of Iba1^+^/TGF-β^+^ cells compared to all populations of Iba1^+^ cells within the contused spinal cord was lower when compared to intact spinal cords. At the same time, the number of Iba1^+^/CD40^+^cells and their percentage ratio to all population of Iba1^+^cells was increased by 60 dpi in the white matter of injured compared to intact spinal cords, while there was no significant difference between the experimental groups with SCI of different degrees of severity. These findings may indirectly indicate normalization or decreased neuroprotective microglia activation in the chronic period after injury (60 dpi), as well as ongoing activation of neurotoxic microglia in the white matter of the injured spinal cord.

Unfortunately, our study was limited by immunohistochemical analysis of different populations of microglia/macrophages within the intact and injured spinal cord only in the chronic period (60 dpi). Therefore, to fully conclude whether the behavior of microglia can affect the severity of SCI and determine the outcome of this disease, further studies of microglia and infiltrating macrophage populations in the acute and subacute periods of SCI are required. In conclusion, our obtained data have potential implications since they support a role of microglia activation affecting the severity of SCI and warrant further investigation of first described annular-shaped microglia to determine their value in the treatment and management of SCI.

## Data Availability Statement

The original contributions presented in the study are included in the article/Supplementary Material, further inquiries can be directed to the corresponding author/s.

## Ethics Statement

The animal study was reviewed and approved by the Kazan Federal University Animal Care and Use Committee (Permit Number: 2 dated on May 5, 2015).

## Author Contributions

EA contributed to immunohistochemistry, obtaining homogenates of spinal cord tissue, and cytokine assay. AT contributed to immunohistochemistry, cDNA synthesis and RT-PCR, and study of microglia morphology. DS contributed to confocal microscopy and statistical analysis. AK contributed to SCI and post-surgical care and behavioral, histological assessment. SA contributed to immunoelectron microscopy. ARo contributed to electrophysiological studies. YM, ARi, and VJ contributed to development of a research plan and participation in the writing of the article. All authors contributed to the article and approved the submitted version.

## Conflict of Interest

The authors declare that the research was conducted in the absence of any commercial or financial relationships that could be construed as a potential conflict of interest.

## Publisher’s Note

All claims expressed in this article are solely those of the authors and do not necessarily represent those of their affiliated organizations, or those of the publisher, the editors and the reviewers. Any product that may be evaluated in this article, or claim that may be made by its manufacturer, is not guaranteed or endorsed by the publisher.
