# Supplementary material for: Increasing Severity of Spinal Cord Injury Results in Microglia/Macrophages With Annular-Shaped Morphology and No Change in Expression of CD40 and Tumor Growth Factor-β During the Chronic Post-injury Stage
Source: Front Mol Neurosci. 2022 Feb 24;14:802558. doi: 10.3389/fnmol.2021.802558 (PMC8908449; doi:10.3389/fnmol.2021.802558)
Supplement: Supplementary file 1 [file Table_1.docx]

**Supplementary Table S1.** Primary and secondary antibodies used in immunofluorescent staining

| **Antibody** | **Host** | **Dilution** | **Source** |
| --- | --- | --- | --- |
| Iba1 | Goat | 1:200 | Abcam |
| CD 40 | Rabbit | 1:200 | Abcam |
| TGF-β | Rabbit | 1:100 | Abcam |
| Anti- goat IgG conjugated with  Alexa 488 | Donkey | 1:100 | Santa Cruz |
| Anti- rabbit IgG conjugated with  Alexa 647 | Donkey | 1:100 | Santa Cruz |
| Anti- goat gold-conjugated secondary antibodies | Donkey | 1:50 | Sigma |

**Supplementary Table S2.** Primers and probes for RT-PCR

| **Primer** | **Nucleotide sequence** |
| --- | --- |
| GAPDH-TM-Forward | ATGACTCTACCCACGGCAAG |
| GAPDH -TM-Reverse | TGGAAGATGGTGATGGGTTT |
| Iba1-TM-Forward | ACCAGCGTCTGAGGAGCTAT |
| Iba1-TM-Reverse | AGGAAGTGCTTGTTGATCCC |
| CD209-TM-Forward | CCTCTCCCAAGTCAGCAGAA |
| CD209-TM-Reverse | CACACCATTTCACACAGCCA |
| TGF-β-TM-Forward | TGCTTCAGCTCCACAGAGAA |
| TGF-β-TM-Reverse | TCCAGGCTCCAAATGTAGGG |
| IL-6-TM-Forward | TGCCTTCTTGGGACTGATGT |
| IL-6-TM-Reverse | CTGGTCTGTTGTGGGTGGTA |
| CD40-TM-Forward | AGTGACAAACAGTACCTCCAAGG |
| CD40-TM-Reverse | TTGATTGAGTTCGCAGTGTCGG |
| TNF-α‎-TM-Forward | CCGTCCCTCTCATACACTGG |
| TNF-α‎-TM-Reverse | GTGCTCATGGTGTCCTTTCC |
| CCL-22-TM-Forward | TTCTTGCTGTGGCACTTCAG |
| CCL-22-TM-Reverse | CTCCTTCACGAAACGTGGTG |
| IL-1β-TM-Forward | GGGATGATGACGACCTGCTA |
| IL-1β-TM-Reverse | TGTCGTTGCTTGTCTCTCCT |
